# Study of Molecular Dimer Morphology Based on Organic Spin Centers: Nitronyl Nitroxide Radicals

**DOI:** 10.3390/molecules29092042

**Published:** 2024-04-28

**Authors:** Dongdong Wei, Yongliang Qin, Zhipeng Xu, Hui Liu, Ranran Chen, Yang Yu, Di Wang

**Affiliations:** 1 School of Materials Science and Chemical Engineering, Anhui Jianzhu University, Hefei 230601, China; 2 Anhui Province Key Laboratory of Condensed Matter Physics at Extreme Conditions, Hefei Institutes of Physical Science, Chinese Academy of Sciences, Hefei 230031, China; 3 School of Advanced Manufacturing Engineering, Hefei University, Hefei 230601, China

**Keywords:** dimer, nitronyl nitroxide (NN), spin coupling, magnetism

## Abstract

In this work, in order to investigate the short-range interactions between molecules, the spin-magnetic unit nitronyl nitroxide (NN) was introduced to synthesize self-assembly single radical molecules with hydrogen bond donors and acceptors. The structures and magnetic properties were extensively investigated and characterized by UV-Vis absorption spectroscopy, electron paramagnetic resonance (EPR), and superconducting quantum interference devices (SQUIDs). Interestingly, it was observed that the single molecules can form two different dimers (ring-closed dimer and “L”-type dimer) in different solvents, due to hydrogen bonding, when using EPR to track the molecular spin interactions. Both dimers exhibit ferromagnetic properties (for ring-closed dimer, J/k_B_ = 0.18 K and ΔE_S−T_ = 0.0071 kcal/mol; for “L”-type dimer, the values were J/k_B_ = 9.26 K and ΔE_S−T_ = 0.037 kcal/mol). In addition, the morphologies of the fibers formed by the two dimers were characterized by transmission electron microscopy (TEM) and atomic force microscopy (AFM).

## 1. Introduction

Organic magnetic materials have long been a subject of interest in the field of magnetic materials due to their lightweight nature, versatile structure, ease of processing and synthesis, and simple fabrication [1,2,3]. In the design of organic magnetic materials, nitronyl nitroxide (NN) radicals have been widely chosen for their unique electron-discrete structure and excellent thermodynamic stability [4,5]. Previous studies have extensively investigated the intramolecular interactions of double radicals and the ferromagnetic or antiferromagnetic magnetism between the spin centers within the molecules [3,6]. Various bridging units, such as pyrene, triptycene, phenylene, spirobifluorene, anthracene, and biphenyl, have been employed to explore the coupling between the double spin centers [7,8,9,10,11,12,13]. These studies have examined the magnetic properties of nitronyl nitroxide radicals, verdazyl radicals [14,15], and TEMPO radicals [16,17,18]. The magnetic field variations caused by different conjugated structures have been extensively studied, while relatively few reports exist regarding the magnetic field changes induced by spin center spatial coupling (i.e., through spatial interactions rather than chemical bond interactions).

There are numerous methods available for investigating intermolecular interactions. These include various weak interactions, such as hydrogen bonding, π-π stacking, hydrophobic interactions, and electrostatic interactions, which play a crucial role in manipulating the aggregation state of organic molecules [19,20,21,22]. However, achieving a direct and explicit characterization of intermolecular interactions and stacking has always been a challenge. This includes understanding whether they involve long-range self-assembled aggregates, short-range interactions between neighboring molecules, or even the formation of a dimer within such multistage assemblies. In this study, the introduction of the organic magnetic center of NN as a marker was employed. Through the hyperfine splitting of NN by hydrogen atoms in EPR spectra, the dimer configurations of several short-range neighboring molecules were clearly revealed. Moreover, the long-range self-assembly was investigated using TEM and AFM techniques. Here, we synthesized a singular spin-centered molecule using 4-(phenyl-azobenzene)benzoic acid as the foundation. The azobenzene unit served as the light-responsive component, the carboxyl group acted as the hydrogen bond donor, and the nitronyl nitroxide radical functioned as the spin center as well as a hydrogen bonding acceptor. Its assembly form varied across different solvents, including dichloromethane (DCM) and toluene. When exposed to ultraviolet light with a wavelength of 365 nm, the azobenzene unit undergoes a structural change, transitioning from a trans configuration to a cis configuration. Consequently, there is a corresponding change in its absorption spectrum [23,24,25]. Moreover, the resulting cis configuration can be stimulated by visible light or heat, causing it to revert back to the trans state and thereby enabling a photostimulated response [26,27].

**Scheme 1:**
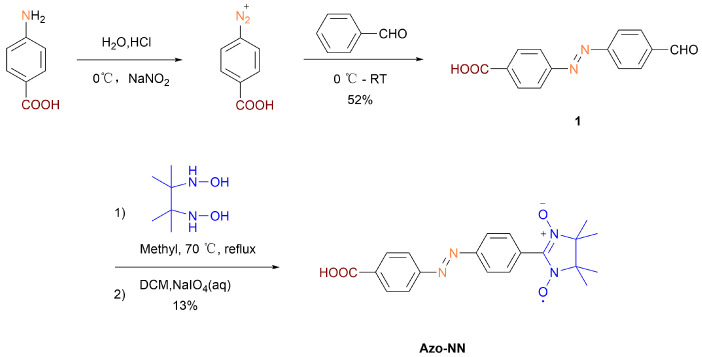
Synthetic routes of Azo-NN.

**Scheme 2:**
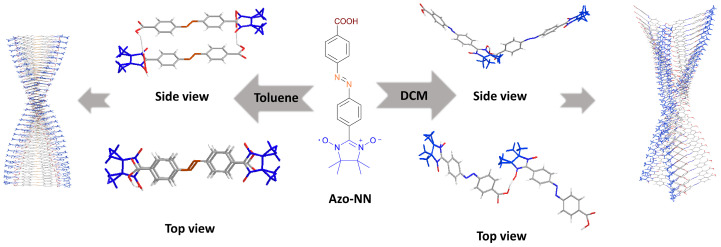
The chemical structure of Azo-NN and its self-assembly, which first forms two different dimers and then two different helical fibers.

In this research, we synthesized a single radical molecule, namely 4-((4-(1,3-dihydroxy-4,4,5,5-tetramethylimidazolin-2-yl)phenyl)diazo)benzoic acid (Azo-NN). This molecule incorporates an NN group at one end of the azobenzene molecule and a carboxyl group at the other end. The structure can undergo photoisomerization under 365 nm UV light (Scheme 1) [7,28], and the carboxyl group can provide a hydrogen bonding site for supramolecular self-assembly bonding (Scheme 2). This approach allows us to explore the multilevel interactions of small organic magnetic molecules in spatial proximity. Through UV characterization and analysis of EPR data, we observed a significant reduction in photocis–trans isomerization of azobenzene after introducing NN to a (*E*)-4-((4-formylphenyl)diazo)benzoic acid assembled into one-dimensional helical fibers due to hydrogen bonding. Prior to the introduction of NN, the complete transition from trans to cis under 365 nm UV illumination occurred in approximately 26 s. However, UV illumination experiments conducted after the introduction of NN showed a prolonged conversion time of 2.5 h under the same UV illumination. We attribute this delay to the hindering effect of hydrogen bonding, which occurs after assembly, on the cis–trans isomeric transition of azobenzene [26]. In the EPR data, the spectrum obtained from the degassed toluene test did not exhibit the classical 1:2:3:2:1 five-line spectrum typically associated with NN [29]. Instead, the Azo-NN spectrum showed hyperfine splitting influenced by the neighboring H nucleus, resulting in a tenfold peak pattern of 1:1:2:2:3:3:2:2:1:1. These data suggest that the assembly of Azo-NN is facilitated by the H⋯O interaction between the H on the carboxyl group and the O in NN [30]. Moreover, the test conducted in DCM exhibited another peak pattern, characterized by an asymmetric ten-line peak. This pattern is indicative of a heterogeneous blend between the spectra of single NN and NN affected by H atoms, suggesting a different form of assembly for Azo-NN in DCM. The two different assembled dimers have a subtle difference in their linking modes, and their long-range assembly, observed under a microscope, shows a similar fibrous structure. Therefore, detecting them using conventional methods is a challenge. However, by analyzing the hyperfine splitting effects on the spin-labeled structure of NN of neighboring hydrogen nuclei, we have gained an additional method to distinguish between the two different dimeric morphologies.

## 2. Results

### 2.1. Optical Properties

**Figure 1:**
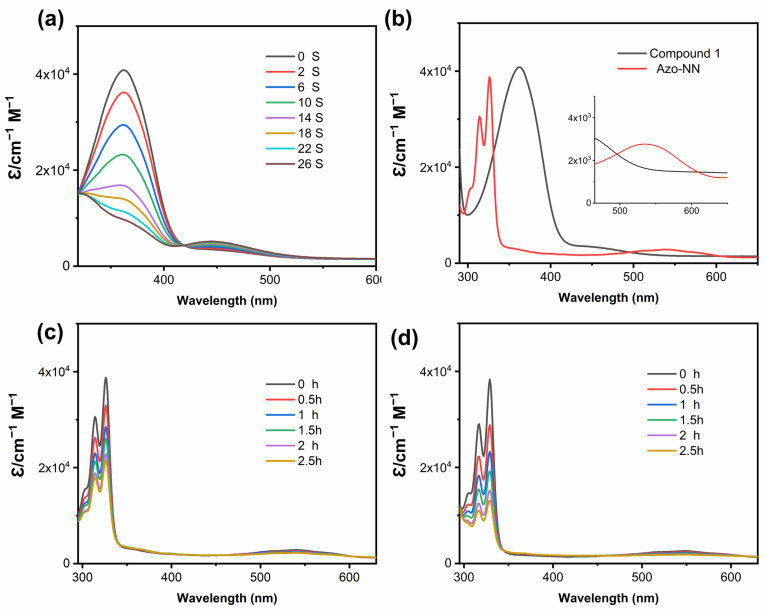
UV absorption spectra at room temperature for samples of 10^−5^ M. (**a**) Compound 1 in THF; (**b**) Azo-NN and Compound 1 in THF (inset—amplification of optical absorption spectra in the visible range from 465 to 650 nm); (**c**) Azo-NN in DCM; and (**d**) Azo-NN in toluene.

UV-visible absorption experiments conducted on compound 1 demonstrated its conversion from a trans to cis structure under 365 nm UV light, as previously reported [27]. The conversion was found to be fully completed in approximately 26 s when using THF as the solvent (Figure 1a). During the process, a gradual decrease in absorption at 365 nm (corresponding to the π–π* transition of the trans isomer) was observed, while a gradual increase in absorbance around 450 nm (corresponding to the *n*–π* transition of the cis isomer) was detected in the spectrum. To synthesize Azo-NN with a photoresponsive molecule, we initially examined its photoisomeric changes in DCM using UV absorption spectra. As shown in Figure 1c, after UV illumination at 365 nm, the absorption of Azo-NN at 330 nm gradually decreased (corresponding to the π–π* transition of the trans isomer), while a gradual increase in absorbance at around 360 nm was observed (corresponding to the *n*–π* jump of the cis isomer) [31]. The apparent absorption at around 550 nm in the visible region indicates the presence of classical NN fine vibrations. Due to the presence of the NN group in the molecule, a partial blue shift was observed in the UV absorption spectrum of the overall molecule [32]. This shift also affected the cis–trans isomeric change in azobenzene, significantly prolonging its rate of change. After several attempts, it was determined that complete conversion required approximately 2.5 h of continuous light exposure. The same results were obtained using toluene as a solvent (Figure 1d). However, when THF was used as a solvent, no data were obtained because Azo-NN is easily destroyed when THF is used as a solvent and when irradiated with UV light at 365 nm.

### 2.2. EPR Spectroscopy

**Figure 2:**
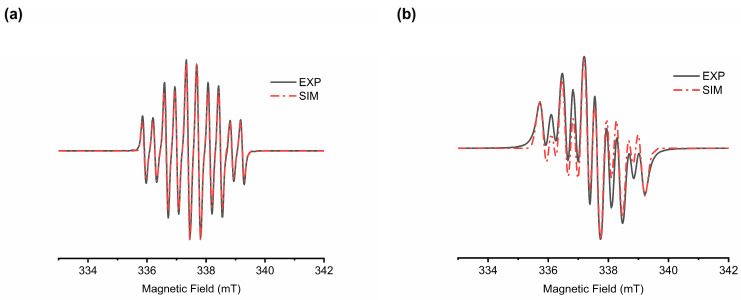
EPR spectra measured at room temperature for samples of 10^−4^ M. (**a**) Azo-NN in toluene and (**b**) Azo-NN in DCM. Black lines are experimental values and red lines are simulated values.

The spectra obtained from our tests in degassed toluene and DCM do not exhibit the classical 1:2:3:2:1 five-line spectra typically observed in NN [9]. Normally, at room temperature, the nitronyl nitroxide radical solutions show five well-resolved lines due to the hyperfine interaction of two equivalent nitrogen nuclei, which corresponds to the typical hyperfine splitting of nitroxide (a_N_ = 0.75 mT). However, in our case, the EPR spectrum suggests that there is an apparent additional exchange interaction between neighboring molecules, as the calculated g-factors and hyperfine coupling constants (hfccs) (a_N_/2) for the biradicals align with values commonly observed in NN in the literature (g = 2.0065, a_N_ = 0.725 mT, a_H_ = 0.355 mT for Azo-NN in toluene; g = 2.0067, a_N_ = 0.705 mT, a_H_ = 0.335 mT for Azo-NN in DCM), confirming the successful synthesis of NN radicals (Figure 2). In contrast, the observed hyperfine splitting of NN by the hydrogen nucleus reveals a ten-line pattern of 1:1:2:2:3:3:2:2:1:1 (Figure 2a). These data indicate that the assembly of Azo-NN is formed through hydrogen bonding between the H on the carboxyl group and the O in NN, resulting in a short contact between the H nucleus and the unpaired single electron, thus causing a corresponding change in the EPR spectrum. Since the assembly is strictly symmetrical and forms a closed ring (Figure 2a), the molecular arrangement suggests that each NN group has formed a hydrogen bond with an H atom on a carboxyl group (we named it the “ring-closed dimer”). On the other hand, the test in DCM reveals another ten-line staggered peak pattern (Figure 2b), which is calculated to be a mixture of single NN and H-atom-influenced NN, indicating an alternative assembly of Azo-NN in DCM. Therefore, this ten-fold peak is the result of the superposition of a set of symmetrical decuplet peaks and a set of symmetrical quintet peaks, to the extent that its EPR spectrum exhibits an asymmetric ten-fold peak. This form can be roughly described as a dimer with the head and tail of the molecule connected (we named it the “L”-type dimer), resulting in a free nitrogen–oxygen radical and an H nucleus, leading to hyperfine splitting. After exposure to 365 nm ultraviolet light, we also conducted EPR testing on the samples using the two solvents mentioned above (Figure S4). We found that the results were not significantly different from those before irradiation, indicating that the cis–trans isomerization of azobenzene did not alter the overall morphology of the dimer. The dimer remained ring-shaped in DCM and retained an “L”-type dimer nitrogen–oxygen radical and a hydrogen bond in the configuration in toluene.

In previous reports, there is no precedent for utilizing EPR to investigate short-range neighboring intermolecular interactions in supramolecular self-assembly. This study represents the first analysis of supramolecular self-assembly using nitroxide radicals and EPR. In the past, supramolecular self-assembly has been limited to probing the assembly of molecules using various microscopes to analyze the arrangement of multiple molecules as well as their micrometer or nanoscale morphology. However, when utilizing SEM or TEM, it becomes challenging to effectively observe the molecular interactions that occur during the formation of dimers and trimers. Our work contributes to the current understanding in this area, as we investigate and analyze the lateral assembly configuration of supramolecular self-assemblies. By employing EPR in conjunction with magnetic studies of nitronyl nitroxide radicals, we delve into the intricate details, offering fresh insights and expanding this field.

### 2.3. Magnetic Properties

**Figure 3:**
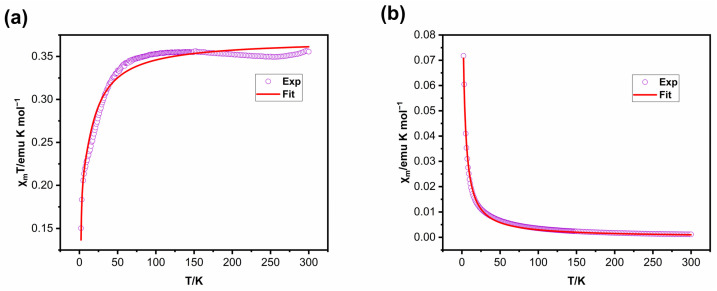
SQUID magnetometry of solid powder radicals: plot of *χ_m_T* vs. *T* and *χ_m_* vs. *T* at H = 10,000 Oe in elevated temperature mode. (**a**) *χ_m_T* vs. *T* and (**b**) *χ_m_* vs. *T*.

To investigate the magnetic properties of the Azo-NN dimers, its SQUID and X-band EPR spectra were further analyzed. The molar magnetization (*χ_m_*) of the Azo-NN powder samples was measured using a SQUID magnetometer at 10,000 Oe across a temperature range of 2–300 K to investigate the magnetic exchange properties of the obtained Azo-NN. To ensure accuracy, the experimental data were corrected by subtracting the measurements obtained from empty samples (Figure 3). The analysis of the experimental data was conducted using Equation (1) based on the Bleaney–Bowers Equation (1).

(1) $$\chi_{m}T=\frac{2N_{A}g^{2}\mu_{B}^{2}/k_{B}}{k_{B}}\frac{T}{T-\Theta }\frac{1}{3+exp(-2J/k_{B}T)}\;$$

The molar magnetization (*χ_m_*) exhibits an increasing trend with temperature in the lower temperature range and eventually reaches a constant value of approximately 0.355 emu K mol^−1^ (Figure 3a) as the temperature increases. This value is close to the theoretical value of 0.375 emu K mol^−1^ expected for a single spin center. The slightly lower molar magnetization strength can be attributed to the influence of intermolecular antiferromagnetism. Figure 3b illustrates the characteristic *χ_m_*-*T* relationship observed for single radical paramagnetism, and the SQUID spectra further confirm the reliability of the obtained molecule. Fitting the experimental data to Equation (1) yields *J*/*k_B_* = 12.86 K, Δ*E_S−T_* = 0.051 kcal/mol, which is close to the calculated DFT of Δ*E_S−T_* = 0.2 kcal/mol.

**Figure 4:**
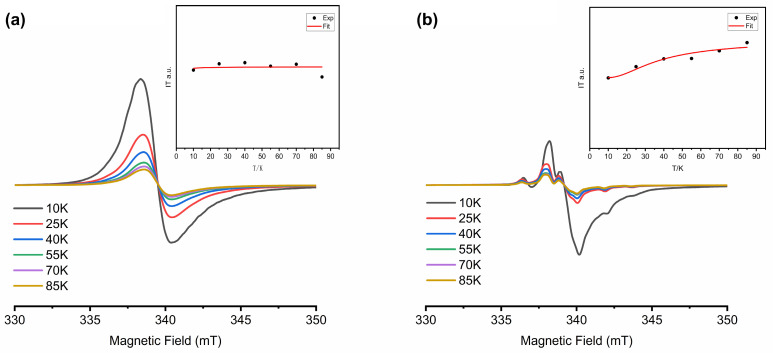
Temperature-dependent EPR spectra from 10 K to 85 K. (**a**) Azo-NN dissolved in DCM of 10^−4^ M and (**b**) Azo-NN dissolved in toluene of 10^−4^ M. Inset—fitting curves according to the Bleaney–Bowers Equation (2).

Temperature-dependent EPR measurements were conducted in the temperature range of 10 K–85 K for both dimers. Due to the dilute solution (~10^−4^ M), the intermolecular coupling can be approximately disregarded. Similarly, in this study, the coupling between dimers can be roughly neglected (Figure 4a,b). The signal intensity gradually decreases with rising temperature. Fitting the data using the Bleaney–Bowers Equation (2) shows

(2) $$IT=C\left[\frac{3\exp\left(2J_{ab}/RT\right)}{1+3\exp\left(2J_{ab}/RT\right)}\right]$$

where *I* is the double integral of the observed EPR spectra, *T* is the temperature (K), *C* is the Curie constant, *J_ab_* is the exchange integral, and the gas constant is *R*. Leading to *J_ab_*/*k_B_* and Δ*E_S−T_* values for ring-closed dimers, specifically *J_ab_*/*k_B_* = 0.18 K and Δ*E_S−T_* = 0.0071 kcal/mol, respectively. For “L”-type dimers, the values were *J_ab_*/*k_B_* = 9.26 K and Δ*E_S−T_* = 0.037 kcal/mol, respectively. To further investigate the coupling mechanism between the two dimers, the EPR spectra of both dimers in frozen solutions were recorded at 100 K (Figure S5). Using the point approximation D = 1.39 × 10^4^ g/r^3^, the average distance (r) between the unpaired electrons can be calculated based on the approximate value of D obtained from the experimental parameters, where g is the g-value of the triplet biradical and D is measured in gausses (Gs) while r is measured in angstroms (Ås). The calculated distances were compared with the distance values obtained from DFT theoretical calculations (defined between the N–O bond centers). In general, a larger value of parameter D indicates a closer spin–spin distance and a stronger interaction coupling within the dimer. For the “L”-type dimer, the parameter 2D was determined to be 75.83 G, and the average distance between the unpaired electrons was found to be 14.31 Å, which is smaller than the DFT theoretical value of 16.61 Å. As for the ring-closed dimer, the coupling between the two NN groups is minimal, resulting in a zero-field splitting signal that approaches that of a single NN group, making it impossible to obtain a value for 2D. Since the D value depends on dipole–dipole interactions between the spins rather than direct exchange interactions, it directly reflects the spatial distance. The finding of a smaller average spin–spin distance in the “L”-type dimer compared to the theoretical calculation suggests the presence of their mutual coupling. Despite the relatively large spatial distance between the two NN groups in the “L”-type dimer, the delocalization of electrons brings the two unpaired electrons closer, enhancing the coupling effect and demonstrating ferromagnetic coupling.

Theoretical calculations were employed to analyze the electron spin density distribution of Azo-NN single radicals and their two self-assembled “double” radicals. The coupling constants between the “double” radicals were calculated using broken symmetry (BS) at the UB3LYP/6-31G(d) level of theory through DFT calculations. The specifics of the calculations can be found in the Supplementary Materials. The magnetic exchange coupling constant (*J*) is determined using the equation proposed by Yamaguchi et al. [33].

(3) $$J=\frac{E_{BS}-E_{T}}{{<S^{2}>}_{T}-{<S^{2}>}_{BS}}$$

**Figure 5:**
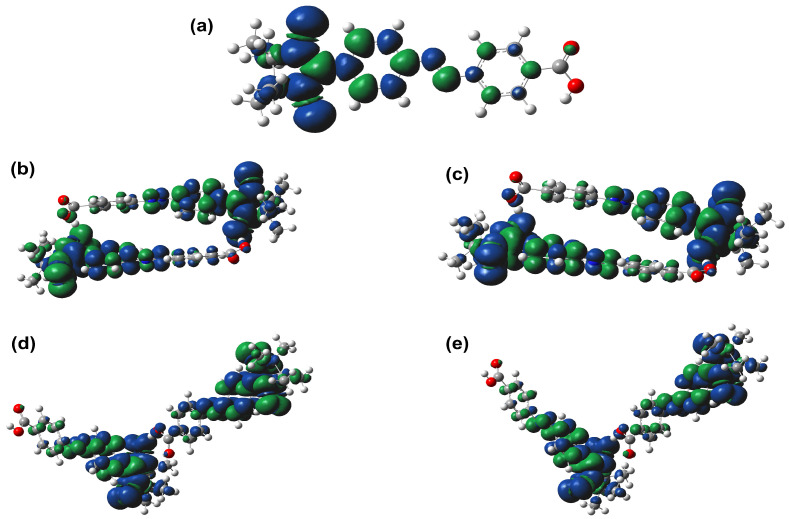
(**a**) Single radical; (**b**) ring-closed dimer singlet state; (**c**) ring-closed dimer triplet state; (**d**) “L”-type dimer singlet state; and (**e**) “L”-type dimer triplet state. Calculated at the UB3LYP/6-31G(d) level of theory. The blue and green surfaces represent the α and β spin densities, respectively.

The total energy of the calculated broken-symmetry (BS) singlet and triplet states is denoted by *E_BS_* and *E_T_*, respectively, while <*S*^2^>*_T_* and <*S*^2^>*_BS_* represent the total spin angular momentum of the calculated symmetry-breaking triplet and singlet states. DFT calculations reveal a similar spin density distribution in the single radical and the two “double” radicals, with a significant spin density concentrated in the benzene ring directly attached to the nitronyl nitroxide radical, and only a minor spin density in the benzene ring farther away from the nitroxide radical (Figure 5). The “double” radicals formed by ring structures exhibit no energy gap (Δ*E_S−T_* = 0) between the singlet and triplet states. In the case of “L”-type dimer, the DFT calculation yields a very weak positive coupling constant (*J_DFT_*/*k_B_* = 50.39 K) and Δ*E_S−T_* = 0.2 kcal/mol. According to the DFT theoretical calculations we conducted, and the data exhibited by the zero-field splitting, we have observed some interesting phenomena. The DFT calculations reveal that there is ferromagnetic coupling between the two NNs in the “L”-type dimer, while there is almost no coupling between the two NNs in the ring-closed dimer. By comparing these findings with our DFT calculations, we have found that the data between the two are consistent. Specifically, for the “L”-type dimer, the ferromagnetic coupling between the two NN groups means that their spins tend to align parallel to each other under interaction, forming a stable magnetic coupling state. On the other hand, for the ring-closed dimer, there is almost no coupling between the two NN groups, indicating that there is no apparent interaction between their spins.

The comparison of SQUID measurements, EPR experiments, and DFT calculations further elucidated our viewpoint. SQUID measurements show a combination of intermolecular and intra-dimer magnetism, while EPR tests predominantly focus on intra-dimer magnetism within the dimer by approximating the elimination of interactions between the dimers. On the other hand, DFT calculations only account for the magnetic properties within a dimer formed by two single molecules joined by hydrogen bonds. Due to the antiferromagnetic interactions between dimers, the DFT-calculated value of *J_DFT_*/k_B_ = 50.39 K is slightly larger than the SQUID and EPR measurement results (Table 1). Within the dimer, a ferromagnetic interaction is observed, which we attribute to the proximity of the two molecules brought about by hydrogen bonding. In both experimental tests and simulation calculations, it was observed that the ferromagnetism of the ring-closed dimer was slightly lower compared to that of the “L”-type dimer. This difference can be attributed to the symmetrical structure of the ring-closed dimer, where two hydrogen bonds are formed with NN, resulting in a more spread out electron spin density. On the other hand, the “L”-type dimer has only one hydrogen atom bonded to NN, causing the electron spin of the connected NN to shift more towards the hydrogen bond. As a result, the “L”-type dimer exhibits a higher degree of ferromagnetism (Figure S7). A half-field forbidden transition (ΔM_s_ = 2) signal is observed in the EPR cryo-freezing spectra of “L”-type dimers dissolved in DCM at 100 K. The EPR resonance of the triplet dimer is split by the magnetic dipole–dipole interaction between unpaired electrons (zero-field splitting—ZFS). Since the ferromagnetic coupling between the two radicals of the ring-closed dimer is weak, its ZFS shows the characteristic peak of a single radical. As for the “L”-type dimer, its ferromagnetic coupling is a bit stronger, and its ZFS presents the characteristic peak of double radicals (Figure S5). After comparing the above aspects, it was found that the dimers linked by hydrogen bonding are ferromagnetic, a finding that differs from some previous reports in the literature. In these reports, the intermolecular or inter-individual magnetic behavior is usually considered to be antiferromagnetic. In contrast, both the “L”-type dimers and the ring-closed dimers studied in this research exhibit ferromagnetic properties.

### 2.4. Characterization of Self-Assembled Structures

**Figure 6:**
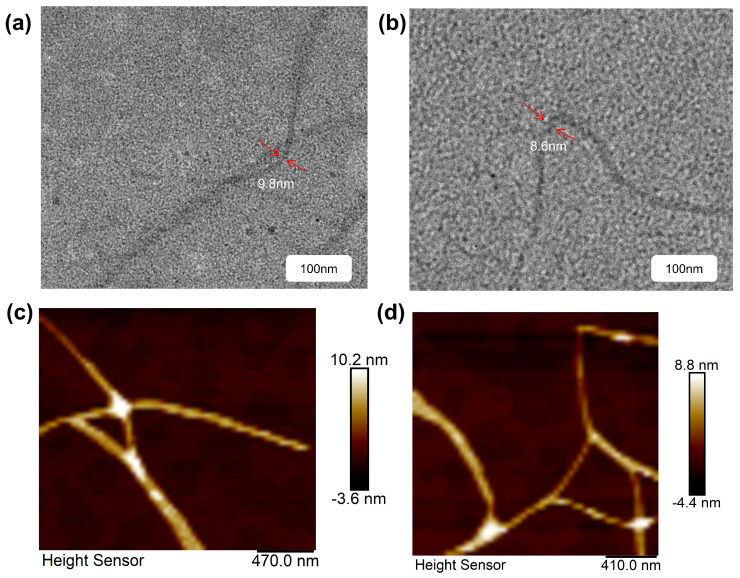
TEM and AFM images of Azo-NN. (**a**) dissolved in DCM; (**b**) dissolved in toluene; (**c**) dissolved in DCM; and (**d**) dissolved in toluene.

The structure of aggregates formed by Azo-NN at a concentration of 10^−4^ M in DCM was observed using TEM (Figure 6a). In DCM, elongated fibrous structures were present. The lengths of these fibers vary, with the smallest fibers having a cross-sectional diameter of about 9.8 nm. These morphological features suggest that the “L”-type dimer conformation of Azo-NN in DCM forms fibers with long-range structure. Similarly, the structure of Azo-NN self-assembled in toluene at a concentration of 10^−4^ M was examined using TEM (Figure 6b). In toluene, the assemblies exhibit a fibrous morphology. However, the fibers have a slightly smaller cross-section than those formed in DCM, with a diameter of approximately 8.6 nm. As the dimer in toluene adopts a closed circular structure, the cross-sectional area of the long-range fiber structure formed is slightly smaller compared to that formed in DCM. The observed self-assembly of fiber formation in toluene is consistent with our previous inference based on EPR spectroscopy. This further suggests that the self-assembly of Azo-NN in DCM adopts a “ring” arrangement. After illumination with UV light at 365 nm, it was disassembled into a spherical shape due to the trans to cis transition of the azobenzene unit, both in DCM and in toluene (Figure S3). For AFM measurements, a DCM or toluene solution of Azo-NN was applied onto the silicon wafer and subsequently analyzed using an AFM. The test results are shown in Figure 6c, and although there are aggregates of various sizes, they are generally 10.2 nm thick. This corresponds to the TEM results we obtained when testing Azo-NN in DCM as a solvent, indicating that Azo-NN formed fibers with a cross-section of approximately 10.2 nm in DCM. And the AFM test in toluene also corroborates its corresponding TEM (Figure 6d), verifying our conjecture.

## 3. Materials and Methods

The synthesis of Azo-NN is depicted in Scheme 1. All reagents necessary for the synthesis were obtained and utilized as per the requirements. The compound 2,3-bis(hydroxyamino)-2,3-dimethylbutane (BHA) was synthesized following a procedure outlined in the literature [34]. Prior to the experiments, all reaction vessels were dried for use and the experiments were carried out under the protection of an argon atmosphere. The specific synthesis details can be found in the Supplementary Materials.

A precursor aldehyde was devised and synthesized for Ullmann condensation with the previously synthesized BHA, followed by oxidation with NaIO_4_ to achieve the desired final product.

Starting from purchased para-aminobenzoic acid, tetrahydrofuran (THF) and ultrapure water were employed as solvents. Sodium nitrite solution and dilute hydrochloric acid were initially added at 0 °C to generate the diazonium salt, followed by adjusting the pH to approximately neutral (pH = 7). Subsequently, benzaldehyde was introduced for the coupling reaction, resulting in the formation of the red-brown compound 1. The characterization of compound 1 was performed using NMR and MS spectroscopy (Figures S1 and S2). The obtained Azo-NN was characterized by UV-VIS and EPR spectroscopy (Figure 1 and Figure 2), confirming the successful synthesis of the target product.

## 4. Conclusions

In conclusion, we successfully synthesized a single radical molecule, which is capable of self-assembly through hydrogen bonds. EPR spectroscopy was used to speculate and confirm the assembly morphology of the dimer, an initiative that has led to a major role for the use of EPR in the field of supramolecular self-assembly. Furthermore, TEM, SQUID, and AFM were used to characterize it and test its corresponding magnetism and self-assembly morphology. Our findings demonstrate the feasibility of employing EPR in the field of supramolecular self-assembly, with the help of the organic magnetic center of NN as a marker, which was ingeniously employed, enabling the detection of dimer assembly morphologies, an area that is often difficult to investigate with conventional electron microscopy.

## Figures and Tables

**Table 1:** Magnetic properties of dimers.

Dimers	*J*/*k_B_* (K) ^a^	*J_ab_*/*k_B_* (K) ^b^	*J_DFT_*/*k_B_* (K) ^c^	Δ*E_S−T_* (kcal/mol) ^a^	Δ*E_S−T_* (kcal/mol) ^b^	Δ*E_S−T_* (kcal/mol) ^c^
Ring-closed dimer	*f*	0.18	0	*f*	0.0071	0
“L”-type dimer	12.86	9.26	50.39	0.051	0.037	0.2

^a^ denotes data from SQUID tests; ^b^ denotes data from EPR tests; ^c^ denotes data from DFT calculations; and *f* denotes not determined.

## Data Availability

Data are contained within the article and Supplementary Materials.

## Supplementary Materials

The following supporting information can be downloaded at https://www.mdpi.com/article/10.3390/molecules29092042/s1. Figure S1. ^1^H NMR spectrum of 1; Figure S2. HR-MS spectrum of 1; Figure S3. TEM image of Azo-NN after 365 nm UV illumination at 10^−4^ M; Figure S4. Electron paramagnetic resonance spectra measured at room temperature; Figure S5. Electron paramagnetic resonance cryogenic freezing spectra at 100K; Figure S6. SQUID magnetometry of solid powder radicals; Figure S7. Spin population of (a) Azo-NN (doublet); (b) “L”-type dimer (triplet) and (c) ring-closed dimer (triplet).
