# Supplementary material for: Study of Molecular Dimer Morphology Based on Organic Spin Centers: Nitronyl Nitroxide Radicals
Source: Molecules. 2024 Apr 28;29(9):2042. doi: 10.3390/molecules29092042 (PMC11085200; doi:10.3390/molecules29092042)
Supplement: Supplementary file 1 [file molecules-29-02042-s001.zip › molecules-2956585-supplementary.pdf]

Supplementary Material

Study of molecular dimer morphology based on organic spin centers: nitronyl nitroxide radicals

Dongdong Wei [1], Yongliang Qin [2], Zhipeng Xu [1], Hui Liu [1], Ranran Chen [1], Yang Yu [3] and Di Wang [1]*

1 School of Materials Science and Chemical Engineering, Anhui Jianzhu University, Hefei 230601, China

2 Anhui Province Key Laboratory of Condensed Matter Physics at Extreme Conditions, Hefei Institutes of Physical Science, Chinese Academy of Sciences, Hefei 230031, China

3 School of Advanced Manufacturing Engineering, Hefei University, Hefei 230601, China

* Correspondence: wangdi@ahjzu.edu.cn

Contents

Materials and Methods	S1
Synthesis	S1
NMR spectra	S3
HR-MS spectra	S4
TEM	S5
EPR spectra	S5
SQUID spectra	S6
Geometry Coordinates	S7

Materials and Methods

Unless otherwise specified, light experiments were conducted using a WFH-204B portable UV detector. The UV-Vis absorption spectra were recorded at room temperature using a CELHXF 300 spectrophotometer. The spectral experiments were conducted at room temperature using toluene and dichloromethane as solvents. NMR spectra were recorded using a 400 MHz Bruker spectrometer with DMSO-d6 as the solvent for proton magnetic resonance (^{1}H NMR) spectra, and the data are reported in ppm relative to the internal standard Me_4Si. High-resolution mass spectra were acquired in ESI mode on the Waters Xevo G2-S QTof instrument, unless otherwise specified. Unless otherwise stated, CW X-band EPR spectra were obtained in an oxygen-free toluene solution at 10^{-4} M. The g-factor corrections were performed using 2,2-diphenyl-1-picrylhydrazyl (g = 2.0037) as a standard, equipped with a frequency counter and temperature control of the liquid helium stream, and simulated with WINEPR SimFonia. Variable temperature magnetization measurements were carried out using the Quantum Design MPMS-XL-7 SQUID Magnetization Meter over a temperature range of 2-300 K with an external magnetic field of 10,000 Oe. Samples were wrapped with approximately 10 mg of Teflon tape and measured in a measurement tube, excluding the background signal from the sample holder with antimagnetic correction. AFM testing was performed on the Bruker Dimension instrument, unless otherwise specified. TEM tests were conducted on the Jem-2100F instrument, unless otherwise specified.

Synthesis

(E)-4-((4-formylphenyl)diazenyl)benzoic acid (1): An aqueous solution (2 mL) of sodium nitrite (0.828 g, 12 mmol) was added dropwise to a magnetically stirred solution of p-aminobenzoic acid (1.370 g, 10 mmol) in dilute hydrochloric acid (3.6 N, 25 mL) at 0 °C. The mixture was stirred at 0 °C for 30 min and the reaction system was added dropwise to a solution of benzaldehyde (1 ml, 10 mmol) after adjusting to pH = 7 with $NaCO_3$ solution at 0 °C. After complete addition, the mixture was reacted at 0 °C for 2 h and then heated to room temperature and stirred for 16 h. The mixture was washed with NaCO3 solution and then acidified with dilute HCl solution. The brown solid was filtered, washed with water and dried. By silica gel column chromatography, (eluent: ethyl acetate/hexane (1:2)) (E)4-((4-formylphenyl) diazenyl) benzoic acid (1.32 g, 52%) was obtained as a brown solid. 1H NMR (400 MHz, DMSO) δ 13.14 (s, 1H), 10.47 (s, 1H), 8.12 (d, J = 8.7 Hz, 2H), 7.87 (dd, J = 13.4, 8.8 Hz, 4H), 6.97 (d, J = 8.9 Hz, 2H).

4-((4-(1-oxyl-3-oxide-4,4,5,5-tetramethylimidazolin-2-yl)phenyl)diazinyl)benzoic acid(Azo-NN): 1)Compound 1 (254 mg, 1 mmol) and 1.5 eq 2,3-Dimethyl-2,3-bis(hydroxylamino)butane (222 mg, 1.5 mmol) were charged into a flask, evacuated and kept under argon. Mixed solvent with MeOH (25 mL) was added into the flask from syringes and was kept argon bubbling for 20 mins. Then the system was heated to 70 ℃ and refluxed for 36 hours. The color of the reaction mixture turned brownish-red. Then the solvent was evaporated and the

white with a little brownish-red solid did not need further purification for synthesis in the next step.

2) Dissolve the product of the previous step in 25 ml of DCM and fill the flask, then place it in an ice-water bath. Take one equivalent of sodium periodate and dissolve it in 15 ml of deionized water. The sodium periodate solution was slowly added dropwise to the reaction system and the reaction was performed in an ice-water bath for about 40 min, and the reaction system changed from brownish red to purple to stop the reaction. Purification was performed using column chromatographic separations (eluent: ethyl acetate/ Dichloromethane (1:1)), and a purple solid was collected.

NMR spectra

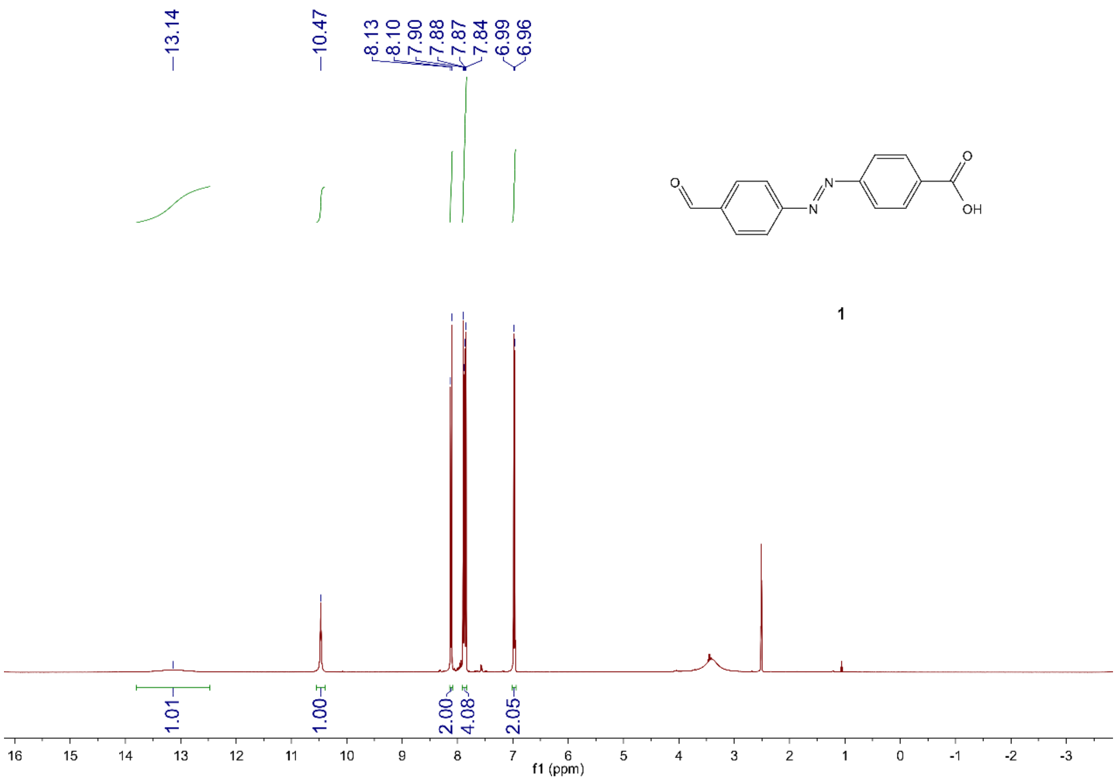

Figure S1. ^{1}H NMR spectrum of **1** (400 MHz, 298 K, DMSO-d6).

HR-MS spectra

Elemental Composition Report

Page 1

Single Mass Analysis

Tolerance = 20.0 PPM / DBE: min = -1.5, max = 50.0
Element prediction: Off
Number of isotope peaks used for i-FIT = 3

Monoisotopic Mass, Even Electron Ions
268 formula(e) evaluated with 1 results within limits (up to 50 closest results for each mass)
Elements Used:
C: 14-14 H: 11-11 N: 0-100 O: 0-100 Na: 0-1
4
230624-11-1 16 (0.187)

1: TOF MS ES+
4.00e+001

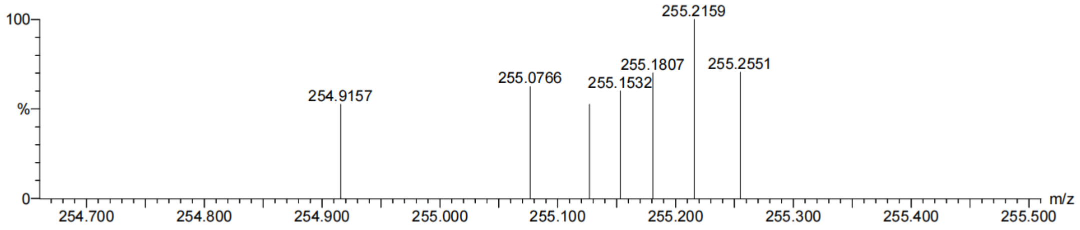

Minimum:				-1.5				
Maximum:		5.0	20.0	50.0				
Mass	Calc. Mass	mDa	PPM	DBE	i-FIT	Norm	Conf(%)	Formula
255.0766	255.0770	-0.4	-1.6	10.5	45.4	n/a	n/a	C14 H11 N2 O3

Figure S2. HR-MS spectrum of **1**

TEM

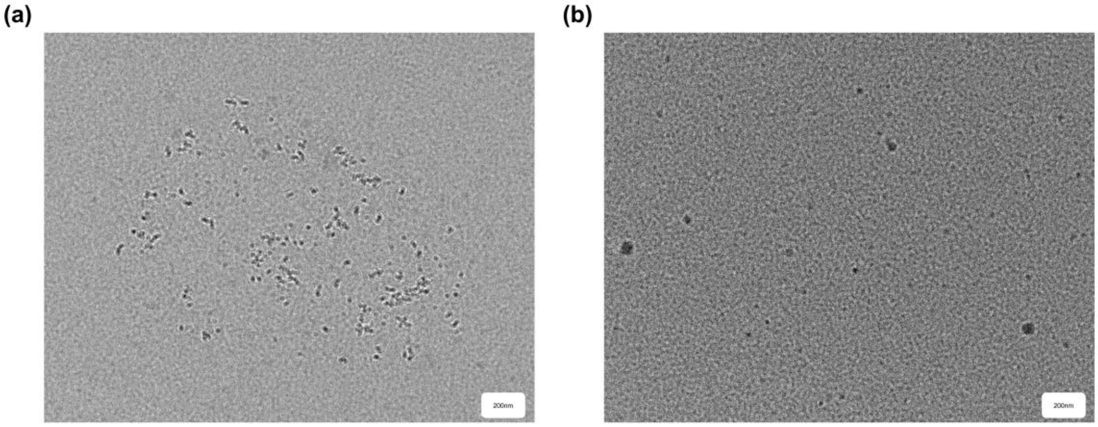

Figure S3. TEM image of **Azo-NN** after 365 nm UV illumination at 10^{-4} M, (a) dissolved in dichloromethane; and (b) dissolved in toluene.

EPR spectra

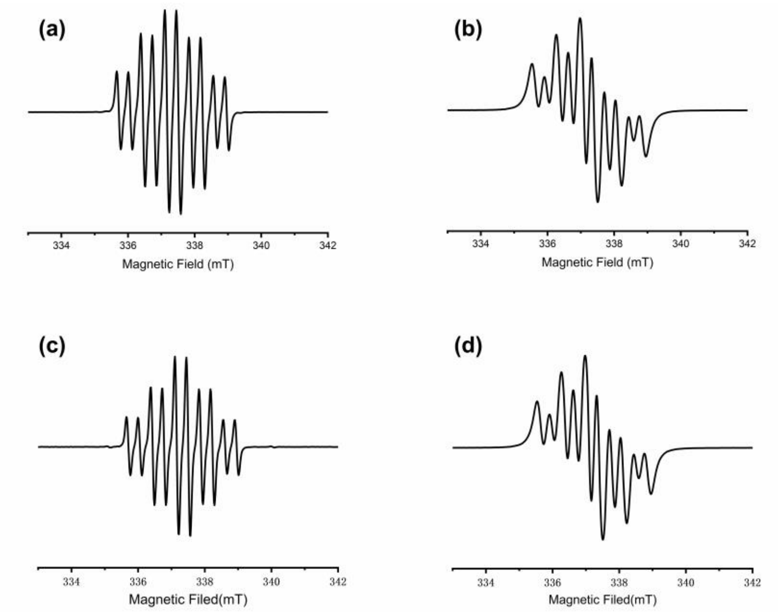

Figure S4. Electron paramagnetic resonance spectra measured at room temperature. (a) **Azo-NN** in degassed toluene at 10^{-4} M; (b) **Azo-NN** in degassed DCM at 10^{-4} M; (c) after 2.5 hours of UV illumination at 365nm wavelengths **Azo-NN** in degassed toluene at 10^{-4} M; and (d) after 2.5 hours of UV illumination at 365nm wavelengths **Azo-NN** in degassed DCM at 10^{-4} M.

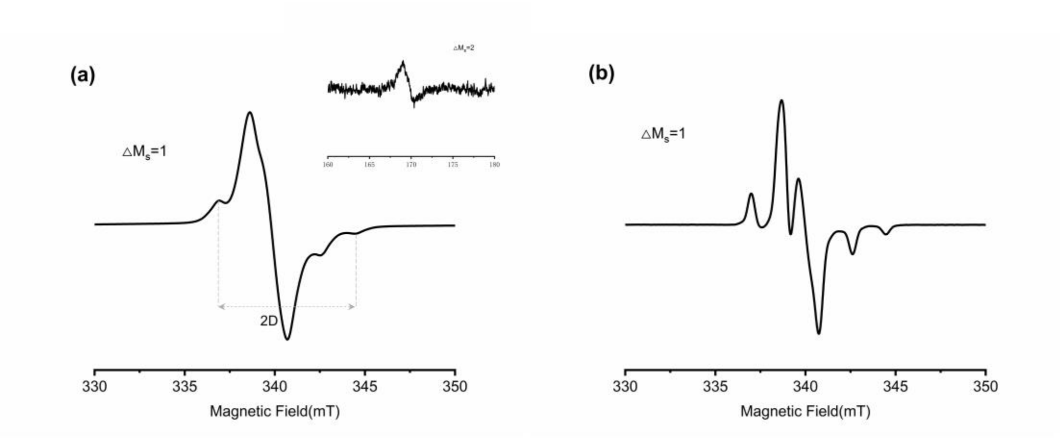

Figure S5. Electron paramagnetic resonance cryogenic freezing spectra at 100K. (a) **Azo-NN** in degassed DCM at 10^{-4} M (inset: forbidden transition of ΔM_s = 2) and (b) Azo-NN in degassed toluene at 10^{-4} M.

SQUID spectra

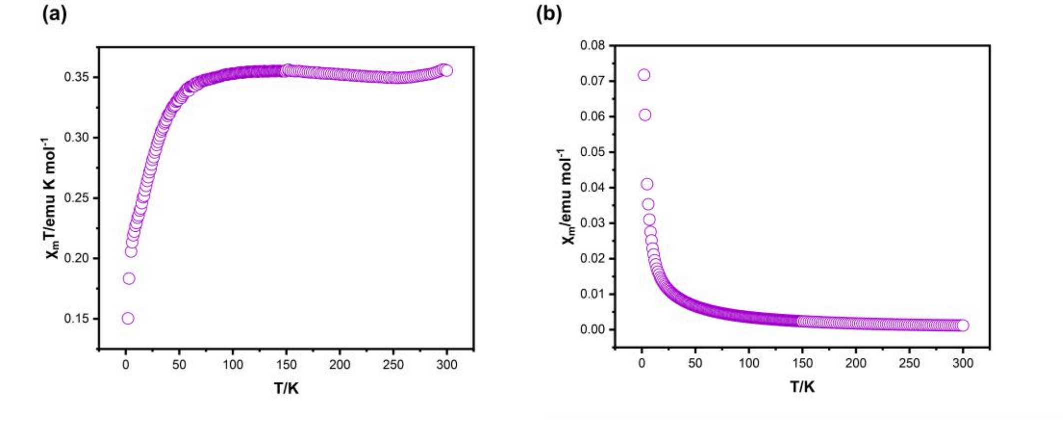

Figure S6. SQUID magnetometry of solid powder radicals. (a) χ_mT vs T and (b) χ_m vs T.

Geometry Coordinates

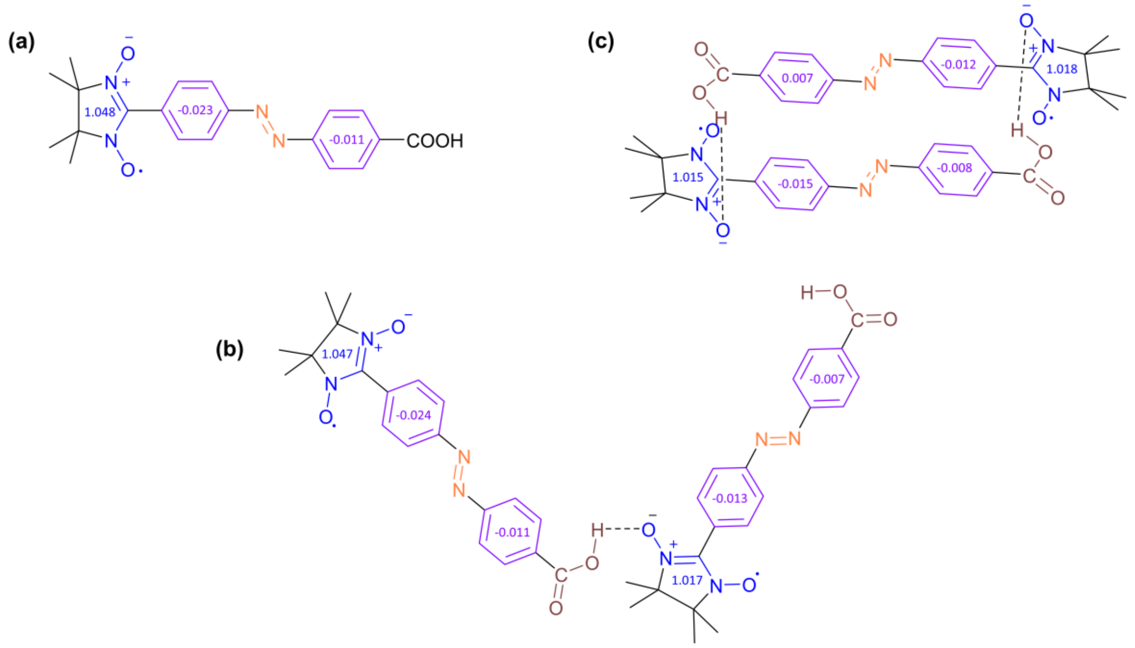

Figure S7. Spin population of (a) Azo-NN (doublet); (b) “L”-type dimer (triplet) and (c) ring-closed dimer (triplet).

Azo-NN(doublet)

Mulliken charges and spin densities with hydrogens summed into heavy atoms:

		1	2
1	C	0.112122	0.083696
2	C	0.046086	-0.068719
3	C	0.033096	0.041810
4	C	0.169558	-0.059335
5	C	0.080649	0.041132
6	C	0.037146	-0.061591
7	N	-0.290030	0.028862
8	N	-0.292169	-0.045379
9	C	0.165653	0.014553
10	C	0.653197	-0.250068
11	C	0.051792	-0.014296
12	C	-0.031794	0.008588
13	C	0.042524	-0.014693
14	C	0.028762	0.008383
15	C	0.105109	-0.014025
16	N	-0.337188	0.271157
17	N	-0.337098	0.272346
18	C	0.135937	-0.016653
19	C	0.134648	-0.016800

20	O	-0.369608	0.376839
21	O	-0.369546	0.377829
22	C	0.089891	0.015754
23	C	0.079994	0.003143
24	C	0.089965	0.015902
25	C	0.079523	0.003122
26	C	0.453988	0.001663
27	O	-0.386533	-0.003092
28	O	-0.175678	-0.000128

Azo-NN(Ring-closed dimer singlet)

Mulliken charges and spin densities with hydrogens summed into heavy atoms:

		1	2
1	C	0.119722	0.062553
2	C	0.051704	-0.046083
3	C	0.036531	0.028983
4	C	0.164509	-0.042572
5	C	0.083674	0.028116
6	C	0.046823	-0.042905
7	N	-0.278119	0.017635
8	N	-0.284564	-0.025199
9	C	0.159961	0.008169
10	C	0.619389	-0.224616
11	C	0.089610	-0.008390
12	C	0.006328	0.005117
13	C	0.034107	-0.008784
14	C	0.034358	0.005055
15	C	0.054345	-0.008223
16	C	0.448844	0.001786
17	N	-0.318818	0.265048
18	N	-0.308790	0.294823
19	C	0.133563	-0.016243
20	C	0.130683	-0.016913
21	O	-0.348625	0.427176
22	O	-0.447652	0.255411
23	C	0.089100	0.003254
24	C	0.096162	0.016032
25	C	0.090937	0.003455
26	C	0.101404	0.016079
27	O	-0.407403	-0.002848
28	O	-0.185057	-0.010886
30	C	0.111380	-0.064827
31	C	0.053746	0.050529
32	C	0.034176	-0.031424

33	C	0.170887	0.045886
34	C	0.082734	-0.030728
35	C	0.041724	0.045166
36	N	-0.284822	-0.021361
37	N	-0.291967	0.029536
38	C	0.160369	-0.009551
39	C	0.642050	0.214694
40	C	0.097035	0.009415
41	C	0.019792	-0.005803
42	C	0.029539	0.010325
43	C	0.018523	-0.005390
44	C	0.039191	0.010002
45	C	0.453909	-0.001659
46	N	-0.324543	-0.269955
47	N	-0.341722	-0.295403
48	C	0.135880	0.016210
49	C	0.144037	0.016539
50	O	-0.343058	-0.438087
51	O	-0.437844	-0.226581
52	C	0.108730	-0.016867
53	C	0.080973	-0.002306
54	C	0.090969	-0.016794
55	C	0.086699	-0.002296
56	O	-0.407084	0.003087
57	O	-0.184028	0.002615

Azo-NN(Ring-closed dimer triplet)

Mulliken charges and spin densities with hydrogens summed into heavy atoms:

		1	2
1	C	0.119741	0.062564
2	C	0.051700	-0.046107
3	C	0.036526	0.028985
4	C	0.164497	-0.042584
5	C	0.083698	0.028129
6	C	0.046774	-0.042899
7	N	-0.278133	0.017621
8	N	-0.284589	-0.025188
9	C	0.159971	0.008094
10	C	0.619437	-0.224629
11	C	0.089638	-0.008250
12	C	0.006328	0.004696
13	C	0.034136	-0.008528
14	C	0.034380	0.004887
15	C	0.054345	-0.008005
16	C	0.448798	0.000122

17	N	-0.318842	0.265022
18	N	-0.308836	0.294881
19	C	0.133577	-0.016236
20	C	0.130687	-0.016913
21	O	-0.348632	0.427124
22	O	-0.447619	0.255472
23	C	0.089096	0.003250
24	C	0.096154	0.016030
25	C	0.090931	0.003453
26	C	0.101391	0.016086
27	O	-0.407387	-0.000409
28	O	-0.185084	0.010694
30	C	0.111378	0.064810
31	C	0.053743	-0.050524
32	C	0.034169	0.031404
33	C	0.170883	-0.045872
34	C	0.082731	0.030710
35	C	0.041736	-0.045159
36	N	-0.284812	0.021278
37	N	-0.291927	-0.029478
38	C	0.160352	0.009404
39	C	0.642079	-0.214691
40	C	0.097034	-0.009239
41	C	0.019814	0.005677
42	C	0.029558	-0.010250
43	C	0.018493	0.006507
44	C	0.039229	-0.009855
45	C	0.453878	0.000753
46	N	-0.324568	0.269984
47	N	-0.341743	0.295339
48	C	0.135875	-0.016216
49	C	0.144044	-0.016533
50	O	-0.343054	0.438105
51	O	-0.437823	0.226610
52	C	0.108734	0.016868
53	C	0.080970	0.002309
54	C	0.090974	0.016791
55	C	0.086677	0.002299
56	O	-0.407085	-0.000850
57	O	-0.184027	0.002457

Azo-NN(“L”-type dimer singlet)

Mulliken charges and spin densities with hydrogens summed into heavy atoms:

		1	2
1	C	0.111165	-0.082487

2	C	0.043295	0.067868
3	C	0.028186	-0.041556
4	C	0.170985	0.059729
5	C	0.079179	-0.040967
6	C	0.035021	0.061334
7	N	-0.292188	-0.029311
8	N	-0.291917	0.043265
9	C	0.166296	-0.014032
10	C	0.653133	0.249146
11	C	0.038329	0.013942
12	C	0.040854	-0.008415
13	C	0.068280	0.014755
14	C	0.010425	-0.008150
15	C	0.090162	0.013649
16	C	0.438063	-0.001294
17	N	-0.337384	-0.272184
18	N	-0.338388	-0.271955
19	C	0.135344	0.016759
20	C	0.135833	0.016752
21	O	-0.371708	-0.374808
22	O	-0.370768	-0.376633
23	C	0.088090	-0.015760
24	C	0.077807	-0.003205
25	C	0.088570	-0.015717
26	C	0.078004	-0.003237
27	O	-0.463200	0.002059
28	O	-0.163589	0.010418
30	C	0.117207	0.066149
31	C	0.048195	-0.050233
32	C	0.036580	0.031555
33	C	0.171467	-0.045512
34	C	0.083914	0.031311
35	C	0.046139	-0.046617
36	N	-0.288660	0.022135
37	N	-0.289947	-0.032033
38	C	0.168278	0.010434
39	C	0.667436	-0.214820
40	C	0.043216	-0.010278
41	C	0.044244	0.006179
42	C	0.067424	-0.010472
43	C	0.023533	0.006032
44	C	0.096570	-0.010019
45	C	0.418108	0.001083
46	N	-0.333705	0.277055

47	N	-0.327481	0.295242
48	C	0.135938	-0.017386
49	C	0.141632	-0.016668
50	O	-0.345230	0.436941
51	O	-0.440866	0.222052
52	C	0.077206	0.003545
53	C	0.103036	0.017153
54	C	0.088972	0.015797
55	C	0.084010	0.003853
56	O	-0.409026	-0.002270
57	O	-0.176071	-0.000175

Azo-NN(“L”-type dimer triplet)

Mulliken charges and spin densities with hydrogens summed into heavy atoms:

		1	2
1	C	0.111258	0.082621
2	C	0.042935	-0.068183
3	C	0.028208	0.041667
4	C	0.170625	-0.059887
5	C	0.079334	0.041084
6	C	0.034791	-0.061565
7	N	-0.291811	0.029346
8	N	-0.292427	-0.043292
9	C	0.166676	0.013984
10	C	0.653130	-0.249395
11	C	0.038398	-0.013930
12	C	0.041380	0.008409
13	C	0.067594	-0.014027
14	C	0.010468	0.008238
15	C	0.090119	-0.013568
16	C	0.438176	0.001310
17	N	-0.337825	0.271751
18	N	-0.337878	0.272431
19	C	0.136183	-0.016662
20	C	0.135469	-0.016806
21	O	-0.371886	0.374908
22	O	-0.370564	0.377190
23	C	0.088222	0.015747
24	C	0.077738	0.003106
25	C	0.088517	0.015843
26	C	0.077652	0.003084
27	O	-0.461651	-0.003058
28	O	-0.164843	0.009117
30	C	0.116930	0.066421
31	C	0.047511	-0.050304

32	C	0.037170	0.031662
33	C	0.171556	-0.045739
34	C	0.084319	0.031445
35	C	0.045945	-0.046761
36	N	-0.288768	0.022240
37	N	-0.289746	-0.032174
38	C	0.168142	0.010476
39	C	0.663803	-0.215461
40	C	0.043309	-0.010323
41	C	0.044224	0.006206
42	C	0.067394	-0.010516
43	C	0.023592	0.006057
44	C	0.096655	-0.010061
45	C	0.418182	0.001089
46	N	-0.332882	0.277013
47	N	-0.327591	0.295007
48	C	0.135326	-0.017252
49	C	0.141988	-0.016527
50	O	-0.345323	0.436500
51	O	-0.439073	0.223991
52	C	0.076964	0.003365
53	C	0.102946	0.017177
54	C	0.088684	0.015719
55	C	0.085818	0.003742
56	O	-0.408987	-0.002279
57	O	-0.176077	-0.000176
